# Analysis of the risk factors for early death due to disease recurrence or progression within 1 year after hepatectomy in patients with hepatocellular carcinoma

**DOI:** 10.1186/1477-7819-10-107

**Published:** 2012-06-14

**Authors:** Toshiya Kamiyama, Kazuaki Nakanishi, Hideki Yokoo, Hirofumi Kamachi, Munenori Tahara, Tatsuhiko Kakisaka, Yosuke Tsuruga, Satoru Todo, Akinobu Taketomi

**Affiliations:** 1 The Department of General Surgery, Hokkaido University Graduate School of Medicine, North 15, West 7, Kita-ku, Sapporo, 060-8638, Japan; 2 The Department of Transplantation Surgery, Hokkaido University Graduate School of Medicine, North 15, West 7, Kita-ku, Sapporo, 060–8638, Japan

**Keywords:** Hepatocellular carcinoma, Hepatectomy, Early death

## Abstract

**Background:**

Liver resection for hepatocellular carcinoma (HCC) has the highest local controllability among all local treatments and results in a good survival rate. However, the recurrence rates of HCC continue to remain high even after curative hepatectomy Moreover, it has been reported that some patients with HCC have an early death due to recurrence. We analyzed the preoperative risk factors for early cancer death.

**Methods:**

Between 1997 and 2009, 521 consecutive patients who underwent hepatectomy for HCC at our center were assigned to group ED (death due to HCC recurrence or progression within 1 year after hepatectomy) and group NED (alive over 1 year after hepatectomy). Risk factors for early cancer death were analyzed.

**Results:**

Group ED included 48 patients, and group NED included 473 patients. The cause of death included cancer progression (150; 78.1%), operation-related (1; 0.5%), hepatic failure (15; 7.8%), and other (26; 13.5%). Between the ED and NED groups, there were significant differences in albumin levels, Child-Pugh classifications, anatomical resections, curability, tumor numbers, tumor sizes, macroscopic vascular invasion (portal vein and hepatic vein), alpha-fetoprotein (AFP) levels, AFP-L3 levels, protein induced by vitamin K absence or antagonism factor II (PIVKA-II) levels, differentiation, microscopic portal vein invasion, microscopic hepatic vein invasion, and distant metastasis by univariate analysis. Multivariate analysis identified specific risk factors, such as AFP level > 1,000 ng/ml, tumor number ≥ 4, tumor size ≥ 5 cm, poor differentiation, and portal vein invasion. With respect to the preoperative risk factors such as AFP level, tumor number, and tumor size, 3 (1.1%) of 280 patients with no risk factors, 12 (7.8%) of 153 patients with 1 risk factor, 24 (32.9%) of 73 patients with 2 factors, and 9 (60.0%) of 15 patients with 3 risk factors died within 1 year of hepatectomy (*p* < 0.0001).

**Conclusions:**

Hepatectomy should be judiciously selected for patients with AFP level > 1,000 ng/ml, tumor number ≥ 4, and tumor size ≥ 5 cm, because patients with these preoperative risk factors tend to die within 1 year after hepatectomy; these patients might be better treated with other therapy.

## Background

Liver resection for the treatment of hepatocellular carcinoma (HCC) has the highest controllability among all local treatments and results in a good survival rate [1,2]. However, recurrence rates remain high and are the main cause of early death even after curative hepatectomy [3]. Moreover, it has been reported that some patients with HCC have an early death due to recurrence [4]. In the remnant liver after hepatectomy, tumor recurrence is recognized as intrahepatic metastasis caused by dissemination of cells in the portal vein or metachronous multicentric hepatocarcinogenesis [5]. The risk factors for early recurrence are reported to be related to tumor cell dissemination due to tumor characteristics such as vascular invasion [6,7] and intrahepatic metastasis [8]. Though the two algorithms that were proposed from the Barcelona Clinic Liver Cancer (BCLC) classification [9] and Japanese guideline [10] recommend that multiple HCCs be treated by transcatheter arterial chemoembolization with lipiodol (TACE) or sorafenib, hepatectomy beyond these algorithms was actually performed in the clinical scene. However, the risk factors for early death due to HCC recurrence or progression within 1 year after hepatectomy have not been clearly evaluated [11].

On the other hand, the Sorafenib Hepatocellular Carcinoma Assessment Randomized Protocol (SHARP) trial [12] recently reported the effectiveness of sorafenib in the treatment of advanced HCC. In this report, median overall survival was 10.7 months in the sorafenib group and 7.9 months in the placebo group. If patients have an early death within 1 year due to recurrence after hepatectomy, there might be no benefit of hepatectomy compared to sorafenib. Therefore, the risk factors for early death within 1 year after hepatectomy due to HCC recurrence or progression should be evaluated, and the appropriateness of hepatectomy for patients with advanced HCC should be investigated.

To identify the risk factors related to early death after hepatectomy, we analyzed the outcomes of 521 consecutive patients who underwent primary hepatectomy for HCC at our center.

## Methods

### Patients

Between January 1997 and May 2009, 521 consecutive patients underwent hepatectomy for HCC at our center. These patients were followed for at least 1 year, and then assigned to group ED (death due to HCC recurrence or progression within 1 year after hepatectomy) or group NED (alive over 1 year after hepatectomy). The resulting ED group included 48 (9.2%) patients, and the resulting NED group included 465 (89.3%) patients. Of all 521 patients, 8 (1.5%) patients who died of liver failure, other causes, and postoperative complications within 1 year after hepatectomy were excluded from group ED and NED. The mean age of 513 patients of group ED and NED was 61.3 years, with a range of 18–87 years. Of the 513 patients, 427 (83.2%) were male and 86 (16.8%) were female, 221 (43.1%) were hepatitis B virus surface antigen-positive, 189 (36.8%) were hepatitis C virus antibody-positive, and 175 (34.1%) had cirrhosis. At least 2 weeks before hepatectomy, imaging studies were performed and preoperative serum alpha-fetoprotein (AFP), *Lens culinaris* agglutinin-reactive fraction of alpha-fetoprotein (AFP-L3), and protein induced by vitamin K absence or antagonism factor II (PIVKA-II) levels were simultaneously measured using standard methods. Among the 513 patients, 499 (97.3%) were categorized as Child-Pugh class A (Table 1). The patients were followed up for a median of 84.2 months (range, 12.5–165.0 months). This study was approved by the Institutional Review Board of the Hokkaido University, School of Advanced Medicine.

**Table 1:** Univariate analysis of the risk factors of death from cancer progression within 1 year after hepatectomy

		**Group ED (*n* = 48)**	**Group NED (*n* = 465)**	***p*****-value**
Sex	Male	40	387	
	Female	8	78	0.9849
Age	<60	24	211	
	60 ≤	24	254	0.5405
HBV	+	26	195	
	-	22	270	0.1033
HCV	+	14	175	
	-	34	290	0.2469
Albumin	<4	33	214	
(g/dl)	4 ≤	15	251	0.0027
Total bilirubin	<0.8	32	283	
(mg/dl)	0.8 ≤	16	182	0.4314
ICGR15 (%)	<15	27	250	
	15 ≤	21	215	0.7421
Child-Pugh	A	42	457	
	B	6	8	<0.0001
AFP (ng/ml)	≤200	15	355	
	200<, ≤1,000	5	37	
	1,000<	28	73	<0.0001
AFP-L3 (%)	≤15	23	334	
	15% < 40<	5	49	
	40<	20	83	0.0002
PIVKA-II (mAU/ml)	≤100	10	258	
	100<, ≤1,000	8	93	
	1,000<	30	114	<0.0001
Liver cirrhosis	Present	17	158	
	Absent	31	307	0.8414
Curability	R0 R1	40	443	
	R2	8	22	0.0008
Anatomical resection	Yes	42	326	
	No	6	139	0.0108
Tumor number	1	16	321	
	2, 3	11	113	
	4 ≤	21	31	<0.0001
Tumor size	≤2 cm	4	64	
	2-5 cm	6	254	
	5 cm ≤	38	147	<0.0001
Macroscopic vascular invasion	Absent	28	440	
(portal vein, hepatic vein)	Present	20	25	<0.0001
Differentiation	Well	0	50	
	Moderate	19	308	
	Poor	29	92	
	Necrosis	0	15	<0.0001
Microscopic portal vein invasion	vp0	10	369	
	vp1	13	60	
	vp2	7	15	
	vp3	12	15	
	vp4	6	6	<0.0001
Microscopic hepatic vein invasion	vv0	28	439	
	vv1	8	12	
	vv2	9	10	
	vv3	3	4	<0.0001
Distant metastasis	Absent	43	459	
	Present	5	6	<0.0001

HCC: hepatocellular carcinoma.

NED: alive 1 year after hepatectomy.

ED: death due to HCC recurrence or progression within 1 year after hepatectomy.

HBV: hepatitis B virus s antigen.

HCV: anti-hepatitis C virus antibody.

ICGR15: indocyanin green retention rate at 15 min.

AFP: alpha-fetoprotein.

AFP-L3: *Lens culinaris* agglutinin-reactive fraction of alpha-fetoprotein.

PIVKA-II: protein induced by vitamin K absence or antagonism factor II.

vp0: no tumor thrombus in the portal vein.

vp1: tumor thrombus distal to the second branches of the portal vein.

vp2: tumor thrombus in the second branches of the portal vein.

vp3: tumor thrombus in the first branch of the portal vein.

vp4: tumor thrombus extension to the trunk or the opposite side branch of the portal vein.

vv0: no tumor thrombus in the hepatic vein.

vv1: tumor thrombus in a branch of the hepatic vein.

vv2: tumor thrombus in the right, middle, or left hepatic vein trunk or the short hepatic vein.

vv3: tumor thrombus to the inferior vena cava.

### Hepatectomy

Anatomical resection is defined as a resection in which lesion(s) are completely removed anatomically on the basis of Couinaud’s classification (segmentectomy, sectionectomy, and hemihepatectomy or extended hemihepatectomy) in patients with sufficient functional reserve. Non-anatomical partial resection was performed as a limited resection or tumor enucleation. When R0 resections were performed, the resection surface was found to be histologically free of HCC. Indocyanine green retention rates at 15 min (ICGR15) were measured to evaluate liver function reserve, regardless of the presence or absence of cirrhosis.

### HCC recurrence

Every 3 months for the first 2 years after hepatectomy, the patients underwent follow-up evaluations comprising liver function tests, measurements of tumor markers AFP and PIVKA-II, ultrasonography (US), and dynamic computed tomography (CT). After 2 years, routine CT was performed only once every 4 months. If recurrence was suspected, CT and magnetic resonance imaging (MRI) were performed; if necessary, CT during angiography and bone scintigraphy were also performed. This enabled precise diagnoses of the site, number, size, and invasiveness of the recurrent lesions.

### Statistical analysis

Patient survival (PS) rates were determined via the Kaplan-Meier method. Univariate analysis was performed; then multivariate analysis and logistic regression were performed only on significant variables. Statistical analyses (StatView 5.0 for Windows: SAS Institute Inc., Cary, NC) were performed using standard tests (*X*^2^, *t*-test) where appropriate. Significance was defined as *p* < 0.05.

## Results

### Causes of death

**Figure 1:**
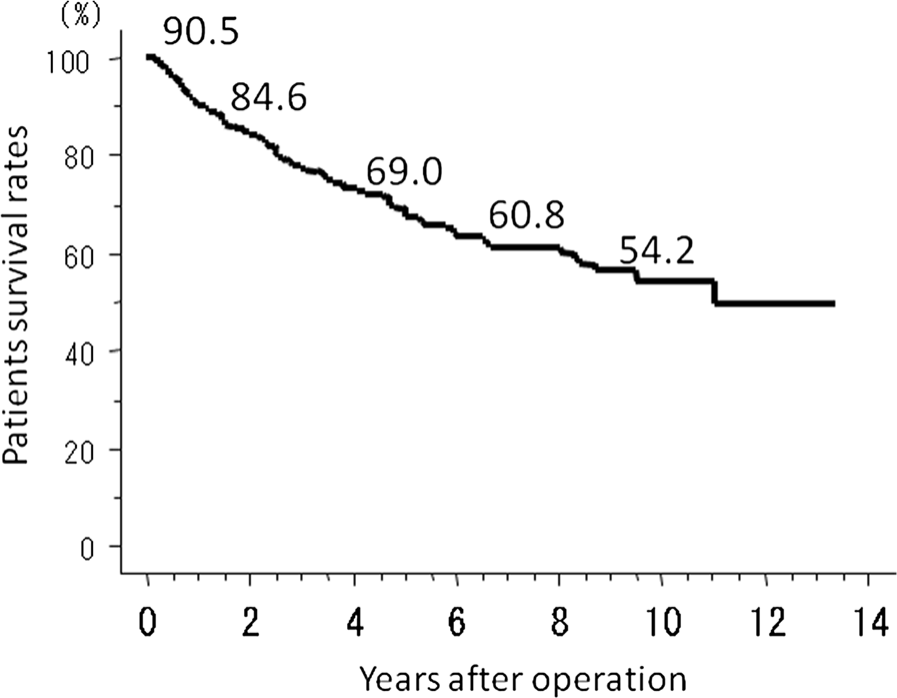
Survivals of all 521 patients at 1, 2, 5, 7, and 10 years were 90.5%, 84.6%, 69.0%, 60.8%, and 54.2%, respectively.

**Figure 2:**
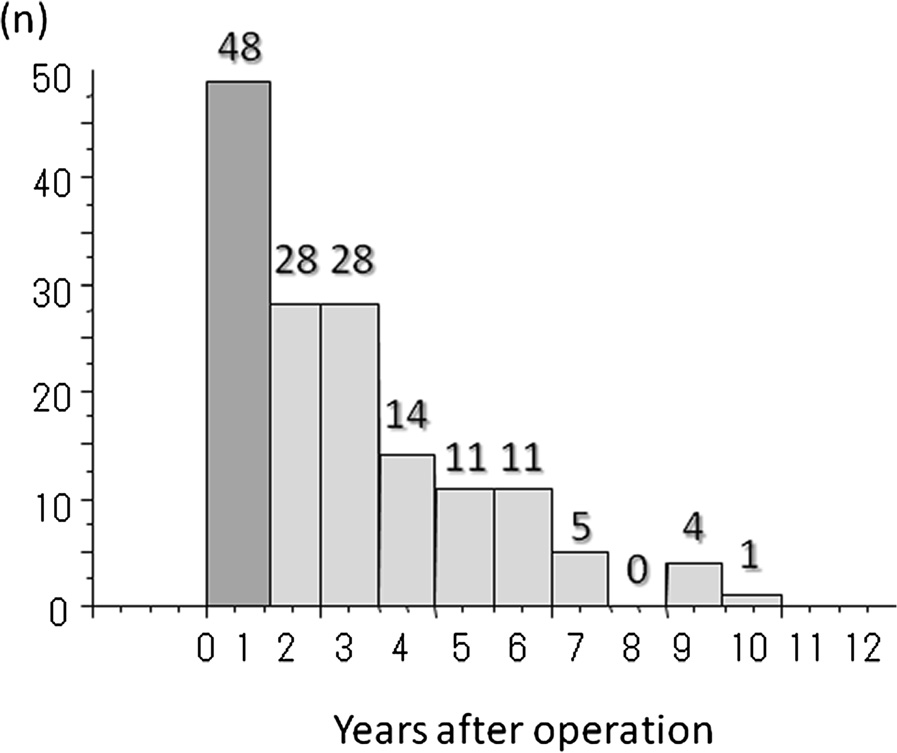
**The number of patients who died of HCC recurrence or progression after hepatectomy.** Of the 150 patients who died of HCC recurrence or progression, 48 patients (32.0%) died within 1 year after hepatectomy.

PS rates (*n* = 521) at 1, 2, 5, and 10 years were 90.5%, 84.6%, 69.0%, and 54.2%, respectively (Figure 1), with 192 deaths (36.9%). The causes of death, whether within 1 year post-hepatectomy or later, included HCC recurrence or progression (*n* = 150; 78.1%), liver failure (*n* = 15; 7.8%), other causes (*n* = 26; 13.5%), and postoperative complications (*n* = 1; 0.5%). Of the 150 patients who died of HCC recurrence or progression, 48 (32.0%) died within 1 year after hepatectomy (Figure 2). The patients who died of liver failure (*n* = 4), other causes (*n* = 3), and postoperative complications (*n* = 1) within 1 year after hepatectomy were excluded from group ED and NED.

### Clinicopathological characteristics and operative variables

Patient characteristics and perioperative outcomes are shown in Table 1. Between the ED and NED groups, there were significant differences in albumin levels, Child-Pugh classifications, anatomical resections, curability, tumor numbers, tumor sizes, macroscopic vascular invasion (portal vein and hepatic vein), AFP levels, AFP-L3 levels, PIVKA-II levels, differentiation, microscopic portal vein invasion, microscopic hepatic vein invasion, and distant metastasis. Tumor-related factors are also shown in Table 1. When the risk factors that were identified as significant by univariate analysis were included in a multivariate analysis via logistic regression, it was found that AFP level, tumor number, tumor size, differentiation, and microscopic portal vein invasion were independent risk factors for early death due to HCC recurrence or progression within 1 year after hepatectomy (Table 2).

**Table 2:** Logistic regression analysis based on univariate analysis of the risk factors of death from cancer progression within 1 year after hepatectomy

**Risk factor**	***p***	**Risk ratio**	**95% CI**
AFP(ng/ml):>1,000(vs. ≤ 200)	0.0079	4.098	1.447-11.628
Tumor number 4 ≤ (vs. 1)	0.0208	3.535	1.206-10.361
Tumor size (cm) 5 ≤ (vs. 2–5)	0.0295	3.687	1.139-11.936
Differentiation poor (vs. moderately)	0.0179	2.8	1.194-6.565
vp1(vs. vp0)	0.0037	5.02	1.691-14.909
vp2(vs. vp0)	0.0034	8.507	2.029-35.667

AFP: alpha-fetoprotein.

vp0: no tumor thrombus in the portal vein.

vp1: tumor thrombus distal to the second branches of the portal vein.

vp2: tumor thrombus in the second branches of the portal vein.

### Risk factors for early death

**Figure 3:**
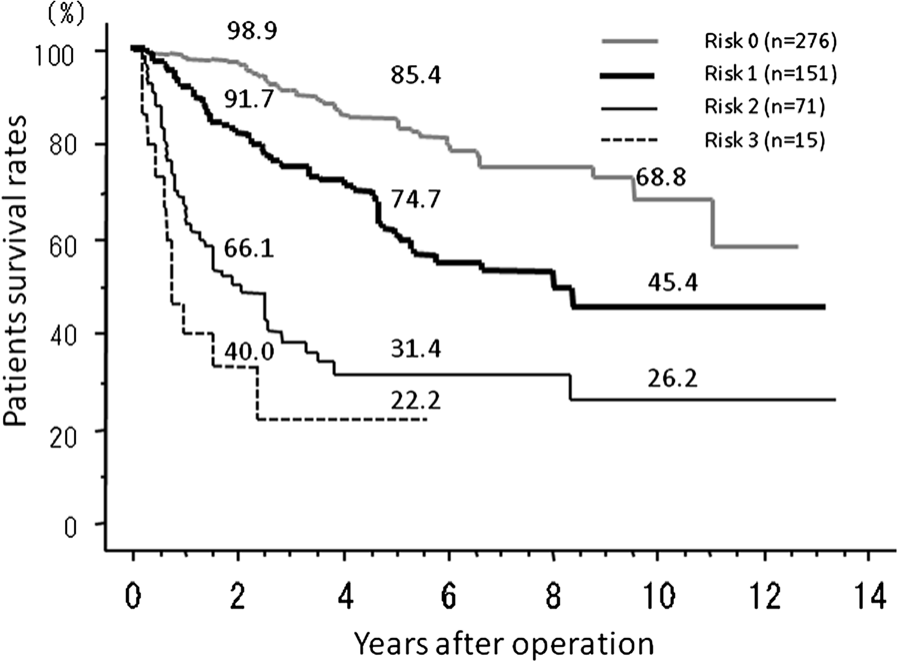
**PS rates for risk 0, risk 1, risk 2, and risk 3 at 1 year were 98.9%, 91.7%, 66.1%, and 40.0%, respectively.** PS rates for risk 0, risk 1, risk 2, and risk 3 at 1 year were 85.4%, 61.5%, 31.4%, and 22.2%, respectively.

Independent, preoperatively evaluable risk factors for early death were identified by multivariate analysis as AFP > 1,000 ng/ml, tumor number ≥ 4, and tumor size ≥ 5 cm. The patients of group ED and NED (*n* = 513) were categorized into three levels of risk: risk 0 if they had no risk factors (*n* = 276), risk 1 if they had any one risk factor (*n* = 151), risk 2 if they had any two risk factors (*n* = 71), and risk 3 if they had all three risk factors (*n* = 15). In risk 0, 3 patients (1.1%), in risk 1, 12 patients (7.9%), in risk 2, and 24 patients (33.8%); in risk 3, 9 patients (60.0%) died within 1 year after hepatectomy (*p* < 0.0001) (Table 3). PS rates for risk 0, risk 1, risk 2, and risk 3 at 1 year were 98.9%, 91.7%, 66.1%, and 40.0%, respectively (Figure 3). Multivariate analysis showed that the risk ratio of risk 1 vs. risk 0 was 7.856, that of risk 2 vs. risk 0 was 46.468, and that of risk 3 vs. risk 0 was 136.5 (Table 3).

**Table 3:** Logistic regression analysis of three risk levels of death from cancer progression within 1 year after hepatectomy

	**No. of patients**	**No. of ED (%)**	**Risk ratio**	**95% CI**
Risk 0	276	3 (1.1)	1	
Risk 1	151	12 (7.9)	7.856	2.181-28.302
Risk 2	71	24 (33.8)	46.468	13.452-160.514
Risk 3	15	9 (60.0)	136.5	29.354-634.752

HCC: hepatocellular carcinoma.

ED: death due to HCC recurrence or progression within 1 year after hepatectomy.

Risk 0: they had no risk factors.

Risk 1: they had any 1 risk factor.

Risk 2: they had any 2 risk factors.

Risk 3: they had all 3 risk factors.

Risk factors: AFP > 1,000 ng/ml, tumor number ≥ 4, and tumor size ≥ 5 cm.

## Discussion

When the patients were categorized by the number of independent, preoperatively evaluable risk factors, the early death rate within 1 year was 60.0% for patients with three risk factors: AFP > 1,000 ng/ml, tumor number ≥ 4, and tumor size ≥ 5 cm, while the early death rate was 1.1% for patients with no risk factors. Therefore, the appropriateness of hepatectomy for HCC should be carefully examined for patients who have large and multiple HCC with high AFP levels; these patients might be better treated with other therapeutic options, such as TACE or sorafenib.

Early recurrence is the main cause of early death within 1 year after hepatectomy. The risk factors for early recurrence are reported to be related to tumor cell dissemination due to tumor characteristics such as vascular invasion [6,7] and intrahepatic metastasis [8]. Because these factors are diagnosed only by postoperative pathological examination, preoperatively evaluable factors are necessary to decide the appropriateness of hepatectomy in advanced HCC. Among preoperative risk factors, an HCC tumor larger than 5 cm is reported to be an important indicator of a high risk of recurrence after resection [13] and has a higher incidence of intrahepatic metastasis and portal venous invasion [14,15]. Therefore, it is believed that an HCC tumor larger than 5 cm has high malignant potential. In this study and another report [11], tumor size ≥ 5 cm reflected this high malignant potential and was selected as an independent risk factor for early death due to HCC recurrence or progression within 1 year after hepatectomy.

Multivariate analysis also shows that tumor number is an important predictor of recurrence. Lai et al. [16] reported that the presence of multiple nodules was the most powerful predictor of both long-term survival and tumor recurrence. Because multiple HCC originates from disseminated cancer cells and not from multicentric carcinogenesis, multiple HCC is a more aggressive phenotype than solitary HCC. Yang reported that, after resection of solitary large HCC, the clinical and pathological characteristics and outcome are similar to those of small HCC, but are significantly better than those of nodular HCC (node number ≥ 2) [17]. It has also been reported that the expression levels of some human genes closely related to invasion and metastasis were significantly lower in solitary large HCC than in nodular HCC [17,18]. They proposed solitary large HCC as a specific subtype, less malignant than nodular HCC. Moreover, in multiple HCC, it was speculated that latent tumors, intrahepatic micrometastases that might be subsequently found to produce early recurrent tumors, could already be present in the remnant liver at the time of surgery. Therefore, tumor number ≥ 4 was selected in the current study as a significant factor predicting early death after hepatectomy.

In our study, multivariate analysis showed that an AFP level over 1,000 ng/ml was an independent factor related to early death. Previous reports have shown that AFP is an independent predictor of prognosis [19], even in patients who had undergone hepatectomy [20]. High levels of AFP in fully developed HCC or in the serum of the host are associated with more aggressive behavior and increased anaplasis [21]. On the other hand, it is well known that AFP levels may increase in some patients with acute and chronic hepatitis without HCC [22,23] and that elevation of AFP levels correlates with inflammation caused by background diseases and hepatocyte regeneration [24]. However, because the elevation of AFP levels by hepatitis or regeneration is usually not so high, only 200 ng/ml [25], AFP levels over 1,000 ng/ml might specifically indicate tumor malignancy. Yamanaka et al. [26] also reported that the serum AFP value per tumor diameter was the most significant risk factor for early death within 1 year after resection in patients with stage II–III HCC by multivariate analysis.

Given these preoperatively evaluable risk factors, the probability of early death after hepatectomy can be estimated by the number of risk factors. In risk 0, 3 patients (1.1%), in risk 1, 12 patients (7.9%), in risk 2, 24 patients (33.8%), and in risk 3, 9 patients (60.0%) died within 1 year after hepatectomy. The risk ratio of risk 1 vs. risk 0 was 7.856, that of risk 2 vs. risk 0 was 46.468, and that of risk 3 vs. risk 0 was 136.5 by multivariate analysis. PS rates for risk 3 at 1 year were 40.0%, while in the SHARP trial, survival rates at 1 year were 44% in the sorafenib group [12]. Moreover, Takayasu et al. reported that the survival rate at 1 year of patients with ≥ 4 tumors, ≥5.1 cm in diameter was 74% [27]. In this way, because the surgical outcome of patients with all three risk factors was worse than that of the patients treated with sorafenib or TACE, these patients might be better treated with other therapeutic options than hepatectomy for the first line treatment. However, selected patients with risk 1 and 2 who might be beyond BCLC and Japanese algorithms should not be excluded from hepatectomy because of their good outcome: 91.7%, 66.1% at 1 year of PS.

On the other hand, in this study, macroscopic vascular invasion (portal and hepatic veins) was not indicated by multivariate analysis as an independent risk factor related to early death. It has been reported that the prognosis of patients with portal vein tumor thrombus (PVTT) in the main trunk or first branch is very poor; the median survival period of patients with portal thrombosis is only 2.7 months without appropriate treatment [28]. However, recently reported patients showed long-term survival rates when hepatectomy was combined with pre- or postoperative treatment. We reported the efficacy of a combination of hepatectomy and preoperative radiotherapy for PVTT in the main trunk or first branch. The 1-, 3-, and 5-year survival rates in hepatectomized patients with preoperative radiotherapy for PVTT were 100%, 53.3%, and 40.0%, respectively [29]. Minagawa [30] reported that the survival rate of patients with PVTT, including those who underwent hepatic resection with preoperative transcatheter arterial chemoembolization, was 42% at 5 years. Nagano [31] reported that 15 patients with HCC with PVTT were treated with FU arterial infusion and interferon therapy (FAIT) and surgery, and that all the patients (100%) survived over 1 year; without FAIT and surgery, 10 patients (67%) died within 1 year. Therefore, even if patients have HCC with macroscopic vascular invasion, particularly PVTT in the main trunk or first branch, hepatectomy is not contraindicated in these patients when combined with pre- or postoperative treatment. In the patients with risk 0, 1, of 45 patients 19 had macroscopic vascular invasion. Of these 19 patients, only 5 (26.3%) died within 1 year after hepatectomy. In the 26 patients with risk 2, 3, 15 patients (57.7%) died within 1 year after hepatectomy. Concerning Child-Pugh B cirrhosis, the high-risk patients could be also indentified. From these data, though macroscopic vascular invasion and Child-Pugh B cirrhosis were poor prognostic factors, the patients who had these factors did not always die in 1 year after hepatectomy. Using our risk levels, the patients with extremely poor prognosis could be identified from the patients who had poor prognostic factors such as macroscopic vascular invasion or Child-Pugh B. Therefore, concerning risk levels, risk 0 to 3 was very important and useful for predicting the prognosis of patients with HCC who underwent hepatectomy.

## Conclusions

In conclusion, the appropriateness of hepatectomy in the treatment of HCC should be carefully considered when patients have the following preoperative risk factors: AFP > 1,000 ng/ml, tumor number ≥ 4, and/or tumor size ≥ 5 cm; these patients might be better treated with other therapeutic options, i.e., sorafenib and TACE. However, even if patients have HCC with PVTT in the main trunk or first branch, hepatectomy is not contraindicated when combined with pre- or postoperative treatment.

## Abbreviations

HCC, Hepatocellular carcinoma; PS, Patient survival; ICGR15, Indocyanine green retention rate at 15 min; AFP, Alpha-fetoprotein; AFP-L3, Lens culinaris agglutinin-reactive fraction of alpha-fetoprotein-L3 fraction; PIVKA-II, Protein induced by vitamin K absence or antagonism factor II; US, Ultrasonography; CT, Computed tomography; MRI, Magnetic resonance imaging; TACE, Transcatheter arterial chemoembolization.

## Competing interests

The authors declare that they have no competing interests.

## Authors’ contributions

TK designed the research; TK, KN, and HY acquired of the data; TK, KN, HY, HK, TK, YT, ST, and AT analyzed the data; TK wrote the paper. All authors read and approved the final manuscript.
